# A biopsychosocial analysis of supplement purchasing behavior in recreational fitness centers: objective BMI, motivational factors, and sex-specific effects

**DOI:** 10.3389/fspor.2026.1867565

**Published:** 2026-09-01

**Authors:** Csilla Ildikó Filó, Dorottya Tóth, Nikolett Ildikó Tumpek, Pongrác Ács, Bence Cselik

**Affiliations:** 1 Physical Activity Research Group, Szentágothai Research Centre, Faculty of Health Sciences, Institute of Physiotherapy and Sport Science, University of Pécs, Pécs, Hungary; 2 Faculty of Health Sciences, Doctoral School of Health Sciences, University of Pécs, Pécs, Hungary

**Keywords:** BMI, dietary supplements, exercise motivation, fitness centers, purchasing behavior, regression modeling, ROC analysis, sex differences

## Abstract

**Background:**

Dietary supplement use in recreational fitness settings is often interpreted through performance-oriented frameworks, yet little is known about how anthropometric status, behavioral engagement, and internalized exercise motives jointly shape real-world purchasing decisions. We examined whether objectively measured BMI, training frequency, current dieting (any type), sex, and motivational structure predict on-site supplement purchase and per-occasion spending in a large gym population.

**Methods:**

In a cross-sectional sample of *N* = 1,087 fitness center users, height and body weight were measured on site and used to compute BMI, and self-reported training, current dieting (a single yes/no item on following any kind of diet), and purchasing/spending data were collected. Five exercise-related motives underwent exploratory factor analysis, yielding two data-derived dimensions: (F1) broad health–appearance motivation and (F2) muscularity vs. weight-loss polarity. Multivariable logistic regression predicted purchase (yes/no), and linear regression modeled log-transformed spending. Sex interactions and a bootstrap mediation (BMI → F1 → spending) were tested. Model discrimination was evaluated using the area under the receiver operating characteristic curve (AUC)

**Results:**

Higher training frequency (OR = 1.79), dieting (OR = 2.14), male sex (OR = 2.05), and F1 motivation (OR = 1.88) independently predicted greater likelihood of purchase, whereas higher BMI was associated with lower purchase odds (OR = 0.88). A significant Sex × F1 interaction indicated that the F1–purchase association was stronger among women. The purchase model showed good apparent discrimination (AUC ≈ 0.82; estimated on the fitting sample). In contrast, spending per occasion was negatively associated with F1, F2, BMI, and training frequency, suggesting more frequent but smaller transactions among highly motivated exercisers. No indirect BMI → motivation → spending pathway was observed.

**Conclusion:**

In recreational gym users, supplement purchasing reflected anthropometric status, behavioral investment, and internalized exercise motives. More engaged and more motivated exercisers were more likely to purchase but spent less per occasion, consistent with structured, goal-congruent consumption, whereas higher BMI was associated with lower purchase odds.

## 1 Introduction

Regular participation in recreational fitness activities has increased substantially over the past decade, alongside the growing availability and visibility of dietary supplements in commercial gym environments. Recent European market data document this continued expansion: fitness-club membership across Europe rose by approximately 4.7 million (+7.5%), from 62.9 million in 2022 to 67.6 million by the end of 2023, surpassing pre-pandemic levels (1). While supplement consumption has been extensively documented in elite athletic populations—historically exceeding 40%–60% depending on sport type and competitive level (2)—recent syntheses confirm that use among elite athletes remains high, commonly ranging from 40% to 100% depending on sport, competitive level, age, and sex (3). Comparable evidence indicates that supplement use is similarly widespread among recreational exercisers: a systematic review of 24 cross-sectional studies (9,202 gym users) reported a high overall prevalence of use, with men favoring protein and performance-oriented products and women favoring health-oriented supplements (4). This shift reflects the convergence of performance-, appearance-, and health-oriented goals in contemporary fitness culture and underscores the need to better understand the biopsychosocial determinants of supplement purchasing behavior in non-elite settings.

Historically, dietary supplement use has often been interpreted through a performance-enhancement framework. However, in recreational fitness contexts, purchasing decisions appear to arise from a more complex interplay of anthropometric characteristics, behavioral engagement, motivational orientations, and situational influences. From a biopsychosocial perspective, supplement consumption can be conceptualized as a health-related behavior shaped simultaneously by biological status, internalized psychological goals, and environmental affordances. Foundational health behavior theories—including the Health Belief Model (5), the Theory of Planned Behavior (6), and Self-Determination Theory (7)—emphasize the role of perceived health needs, goal internalization, and behavioral regulation in shaping health-related actions, including nutrition-related decision-making. Although these frameworks were developed for broader health behaviors, on-site supplement purchasing is a discretionary, goal-directed nutrition behavior and thus falls within their explanatory scope: perceived health or appearance need (Health Belief Model), the translation of intention and perceived control into action (Theory of Planned Behavior), and the degree to which exercise-related motives are internalized (Self-Determination Theory). We use them here as complementary interpretive lenses rather than as formally tested models, since their constituent constructs were not directly measured.

Anthropometric status, particularly body mass index (BMI), may play a role in shaping exercise-related consumption behaviors. BMI was selected as the anthropometric indicator because it is objective, inexpensive, non-invasive, and highly reproducible, which supports its use in large field samples. We note, however, that BMI cannot distinguish fat mass from lean mass, and that individuals with a similar BMI may pursue markedly different goals—for example weight loss vs. muscle hypertrophy—which makes the interpretation of BMI in fitness populations inherently complex. BMI is therefore used here strictly as a marker of weight status, not as a proxy for body composition, body image, or any psychological construct (8). Body image theories suggest that discrepancies between actual and ideal body states may trigger compensatory or restrictive behavioral strategies (9). Importantly, body image concerns are not a function of BMI and can occur across the whole BMI range. Within gym environments—where muscularity and leanness ideals are highly salient—body image concerns may influence not only exercise participation but also related consumption choices (10, 11).

Beyond anthropometric status, behavioral engagement—most notably training frequency—represents a key determinant of health-related actions. Extensions of the Theory of Planned Behavior suggest that stronger behavioral investment enhances consistency between intentions and enacted behaviors (6). Recreational exercisers who train more frequently may therefore be more likely to adopt complementary practices, such as supplement use, as part of a broader exercise routine. Evidence from intervention studies further suggests that combined exercise–nutrition programs can induce meaningful changes in body composition, physical fitness, and lifestyle-related health indicators (12, 13).

Dieting behavior constitutes another important contextual factor. In the present study, dieting refers broadly to currently following any kind of diet in daily life, without specification of type or purpose (see Methods). Individuals who report following structured diets often pursue explicit dietary goals, within which supplements may function as convenient, goal-congruent tools rather than discretionary products; among gym users, muscle building, weight management, and health improvement are frequently reported motives for supplement use (4). Prior research in both clinical and community-based settings indicates that nutrition-related behaviors are frequently embedded within broader lifestyle management strategies (14, 15). These findings suggest that dieting status may independently predict supplement purchasing behavior in recreational fitness environments.

Motivational regulation further shapes supplement-related decision-making. Self-Determination Theory posits that more internalized and integrated motives are associated with greater behavioral consistency across related domains (7). In fitness contexts, such motives rarely occur in isolation; rather, they form multidimensional motivational profiles that may guide both exercise participation and nutrition-related choices. Empirical studies have demonstrated that exercise-related identity and motivation can mediate psychological outcomes such as social appearance anxiety (16).

Sex differences add an additional layer of complexity. Women are more likely to report integrated appearance–well-being motives and heightened sensitivity to body image–related experiences in gym settings, whereas men more often emphasize muscularity and performance-oriented goals (10, 17, 18). These differences suggest that sex may not only independently influence supplement purchasing behavior but also moderate the relationship between motivational factors and purchasing decisions.

Finally, it is important to distinguish between the likelihood of purchasing supplements and the magnitude of spending per occasion. While motivational and behavioral factors may strongly influence the decision to purchase at all, spending behavior is often more sensitive to situational constraints such as pricing, timing, and contextual cues. Moreover, potential mediation pathways—such as BMI influencing spending indirectly through internalized health–appearance motivation—remain underexplored in recreational fitness populations.

Despite the growing literature on exercise motivation, body image, and health-related behaviors, few studies have simultaneously examined objective anthropometric status, behavioral engagement, dieting practices, motivational structure, sex moderation, and both purchase likelihood and spending magnitude within a unified analytical framework. To address these gaps, the present study investigates the biopsychosocial determinants of on-site supplement purchasing in a large sample of recreational fitness center users. Grounded in behavioral and motivational theory, we advance a set of expectations that combine confirmatory, theory-driven predictions (H1, H2, H4) with exploratory hypotheses (H3, H5–H7) that concern empirically derived motivational dimensions and are therefore examined in a more tentative, hypothesis-generating manner:

H1: Higher training frequency will be positively associated with supplement purchasing.
H2: Individuals currently dieting will show a higher probability of supplement purchasing.
H3 (exploratory): Higher levels of the health–appearance motivation dimension will increase purchase likelihood.
H4: Higher BMI will be associated with a lower likelihood of on-site supplement purchasing.
H5 (exploratory): Sex may moderate the motivation–purchase association.
H6 (exploratory): Motivational and behavioral engagement factors may predict per-occasion spending, with smaller effects than for purchase likelihood.
H7 (exploratory): The association between BMI and supplement spending may be partially mediated by health–appearance motivation.

## 2 Methods

### 2.1 Study design and participants

The study employed a cross-sectional survey design targeting adult members of a commercial recreational fitness chain. Data were collected at two facilities of the same commercial fitness chain (Fitness5) in the South Transdanubian region of south-western Hungary: a large two-floor city-centre club in Pécs and a modern facility of more than 1,300 m^2^ in Szekszárd. Both offer a broad range of premium resistance and cardiovascular equipment and group exercise classes served by qualified personal trainers. The clientele is socioeconomically mixed, comprising university students, working adults, and recreational exercisers; membership is available through individual passes and widely used employer-subsidized multi-facility fitness passes (e.g., AllYouCanMove, MaxiSport Pass), which broadens the demographic reach of the sample. Participants voluntarily completed a comprehensive questionnaire assessing demographic characteristics, objectively measured anthropometric information, training frequency, current dieting, exercise-related motivations, and on-site supplement purchasing and spending behaviors. A total of 1,087 participants provided complete and valid data for the primary analyses.

### 2.2 Measures

#### 2.2.1 Demographics and anthropometrics

Participants reported age and sex. Body weight and height were measured on site by trained staff using standard equipment; BMI (kg/m²) was computed from these measured values rather than from self-report, reducing the reporting bias common in supplement-use surveys.

#### 2.2.2 Training behavior

Training frequency was assessed using a 4-level categorical item (1 = “less often”; 2 = “1–2 times per week”; 4 = “3–4 times per week”; 7 = “daily”), where the numeric codes serve as category anchors approximating weekly session counts; intermediate integers were not response options.

#### 2.2.3 Dieting and nutritional practices

Participants indicated whether they currently followed any form of diet in their daily life (single dichotomous item: “Do you follow any kind of diet?”; 0 = no, 1 = yes). The item did not distinguish among diet types or their underlying purposes (e.g., medically indicated, intolerance-related such as lactose-free, weight-management, or sports-nutrition regimens). Diet type, professional supervision, nutritional knowledge, and exposure to marketing or social media were not assessed; this is addressed in the Limitations.

#### 2.2.4 Protein bar purchase and spending

On-site purchasing was assessed for supplements available at the facility—primarily protein/sport bars and comparable single-serve products sold at reception. Purchase (yes/no) was operationalized using a combined rule: participants were coded as purchasers if (a) their selected items did not include any negating expression (e.g., “I don't buy anything”) and (b) a valid spending category was provided. Spending per purchase was reported in four ordered categories, recoded to midpoints (500, 1,750, 3,750, 6,000 HUF; approximately €1.25, €4.40, €9.40, and €15.00 at the data-collection exchange rate of ∼400 HUF/EUR in autumn 2025) for continuous analysis. Median spending per visit was 500 HUF (≈ €1.25) (IQR: 500–1,750 HUF).

#### 2.2.5 Motivation items

Exercise motivation was measured using five dichotomous indicators (Health, Weight loss, Appearance, Muscle, Stress reduction). These items were factor-analyzed to identify underlying latent dimensions.

### 2.3 Exploratory factor analysis (EFA)

EFA was conducted on the five motivation items using the correlation matrix of standardized item scores. Sampling adequacy was acceptable (KMO = 0.67); Bartlett's test indicated factorability [*χ*²(10) = 508.16, *p* < 0.001]. Eigenvalues suggested a two-factor solution (1.895, 1.034). Factor scores were computed using the regression method. Factor 1 reflected general health–appearance motivation (loadings 0.47–0.76); Factor 2 represented a muscularity vs. weight-loss polarity (loadings 0.62 and −0.76, respectively). Because the factor analysis was based on only five dichotomous indicators, the resulting dimensions (F1, F2) are treated as exploratory, data-derived summaries of co-occurring motives rather than as validated psychological constructs; their labels are descriptive of the loading pattern and should not be read as measured latent traits. These factor scores (F1, F2) were used as predictors in subsequent regression analyses.

### 2.4 Statistical analyses

#### 2.4.1 Logistic regression predicting supplement purchase

A multivariable logistic regression model predicted the binary outcome of supplement purchase. Predictors included BMI, training frequency, diet, sex, the two motivation factors (F1, F2), and sex-by-factor interaction terms. The model was fitted via maximum likelihood. Model performance was evaluated via the area under the receiver operating characteristic curve (AUC). The final model achieved an AUC of 0.819. The AUC was estimated on the same sample used to fit the model; no internal (cross-validation or bootstrap optimism correction) or external validation was performed. The reported value should therefore be interpreted as apparent discrimination and may be optimistic.

#### 2.4.2 Linear regression predicting spending

Spending was analyzed as a continuous outcome using linear regression on log-transformed spending [log(x + 1)] to correct for skewness. Predictors matched the logistic model (excluding interactions unless otherwise noted). Standardized beta coefficients were computed via z-scoring of all predictors and the outcome.

F1 and F2 were both significant negative predictors of spending, consistent with more motivated participants spending less per occasion. This analysis was used to evaluate H6.

#### 2.4.3 Mediation analysis

A mediation model tested whether F1 mediated the association between BMI and spending. Path a (BMI → F1) and path b (F1 → log spending) were estimated using OLS. Indirect effects were computed as the product a × b, and confidence intervals were derived from 5,000 bootstrap samples. The indirect effect was small and not statistically significant (95% CI: −0.00463 to 0.00091).

### 2.5 Model diagnostics

Variance inflation factors (VIF) indicated no problematic multicollinearity (all <5.2). Residual and distributional assumptions for the linear regression were checked; log-transformation improved normality and homoscedasticity. Supplement purchase classification used all available cases, while spending models were restricted to respondents with valid spending reports.

### 2.6 Ethics statement

This study involved research with human participants using anonymous, non-invasive questionnaire data and no medical intervention. It was reviewed and approved by the Regional Research Ethics Committee (Regionális Kutatásetikai Bizottság, RKEB; approval code 28-PTERKEB2025/1; approved 30 January 2026). All procedures were conducted in accordance with the Declaration of Helsinki, and written informed consent was obtained from all participants prior to participation.

## 3 Results

A total of 1,087 recreational fitness center users were included. Participants were on average 29.08 ± 11.42 years old, 40.4% were male, and the mean measured BMI was 23.67 ± 4.03 kg/m^2^. The average training frequency was 3.80 ± 1.56 sessions per week, and 19.8% reported currently following a diet. Supplement purchasing was common (80.7%), with a median per-occasion spending of 500 HUF (≈€1.25) (IQR: 500–1,750 HUF) (Table 1). Full descriptive statistics, including variable coding and units, are provided in Table 1 and are therefore only summarized briefly here.

**Table 1:** Descriptive statistics of the sample.

Variable	Value
Sample size (*N*)	1,087
Male (%)	40.4%
Age, mean ± SD	29.08 ± 11.42
BMI, mean ± SD	23.67 ± 4.03
Training frequency (sessions/week)	3.80 ± 1.56
Dieting (%)	19.8%
Supplement purchase (%)	80.7%
Spending per occasion (median, IQR)	500 Ft (500–1,750)

### 3.1 Psychometric properties of the motivation items

**Figure 1:**
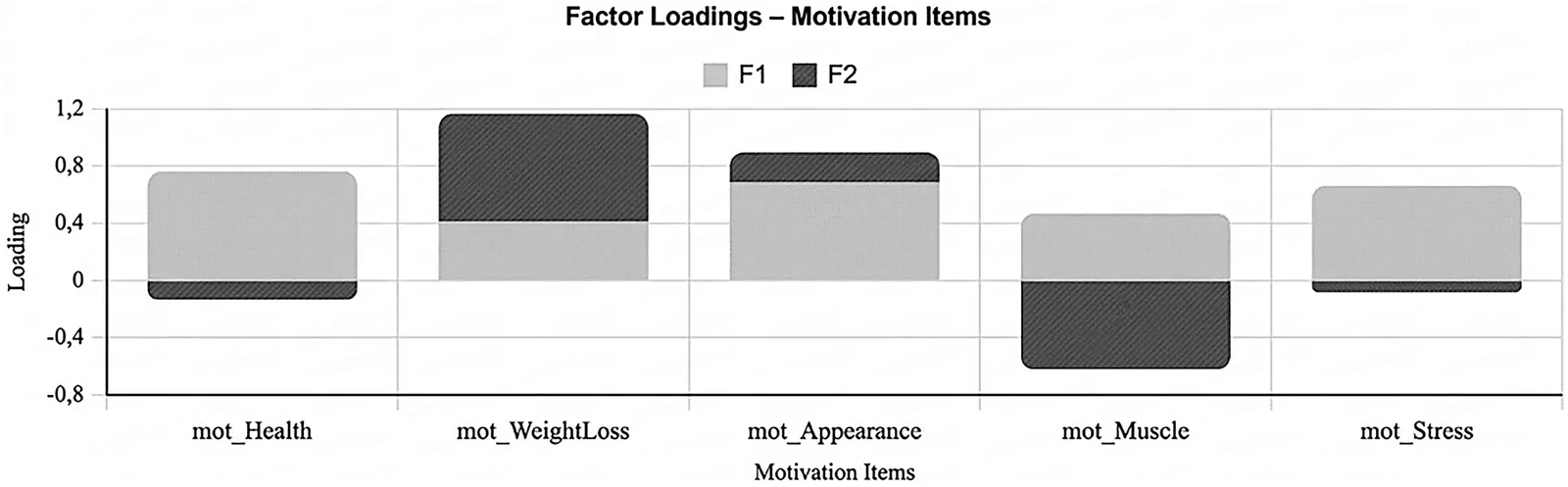
Scree plot of the motivation items.

Exploratory factor analysis confirmed factorability of the five motivation indicators [KMO = 0.67; Bartlett's *χ*^2^(10) = 508.16, *p* < .001]. Two factors exceeded the eigenvalue >1 criterion (1.895; 1.034), supporting a two-factor solution (Figure 1). Factor 1 reflected a broad health–appearance engagement dimension, while Factor 2 represented a muscularity vs. weight-loss polarity (Table 2). As noted in the Methods, these dimensions are treated as exploratory, data-derived summaries rather than validated constructs. These factor scores (F1, F2) were used in tests of H3, H5, H6, and H7.

**Table 2:** Factor loadings for the full-motivation EFA.

Item	F1	F2
Health	0.763	0.135
Weight loss	0.410	−0.759
Appearance	0.692	−0.206
Muscularity	0.472	0.625
Stress reduction	0.665	0.083

The figure displays the eigenvalues of the five motivation indicators derived from the exploratory factor analysis (EFA). The red dashed line indicates the Kaiser criterion (eigenvalue >1). Two components exceeded this threshold, supporting a two-factor structure of the motivation scale.

### 3.2 Predictors of supplement purchase

**Figure 2:**
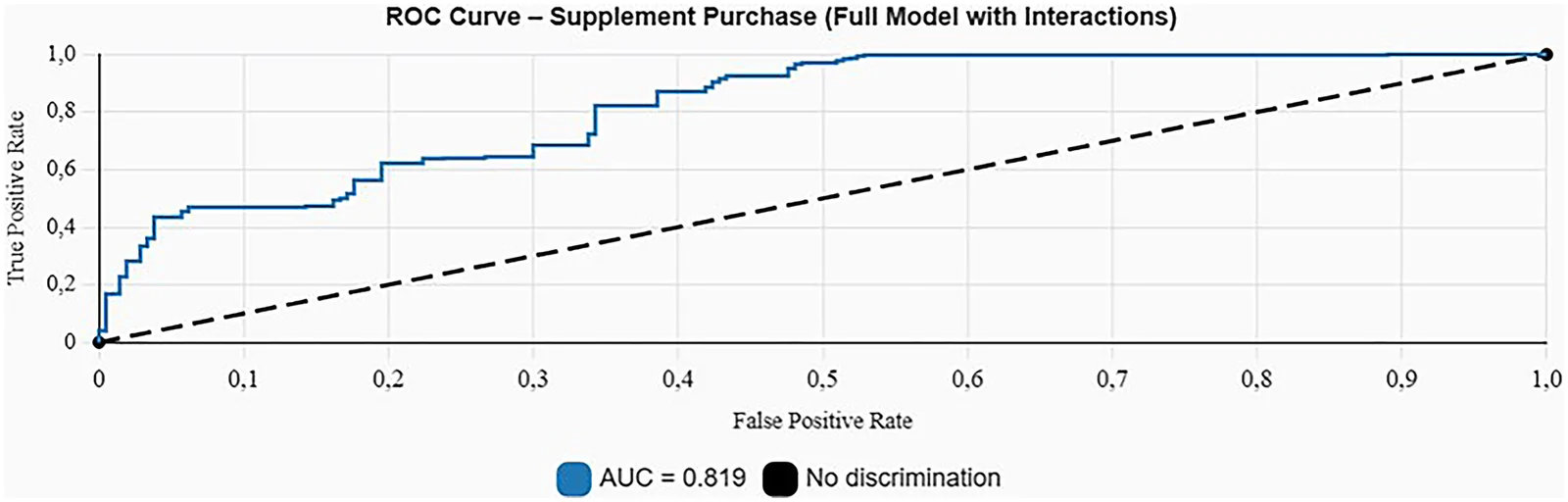
ROC curve for the multivariable logistic regression model predicting supplement purchase.

The multivariable logistic regression model was significant (LLR *p* < .001) and showed good apparent discrimination (AUC = 0.819, estimated on the fitting sample; Figure 2). Higher BMI was associated with a lower likelihood of supplement purchase (OR = 0.875, *p* < .001), whereas higher training frequency (OR = 1.791, *p* < .001) and dieting (OR = 2.137, *p* = .006) predicted higher odds. Men were more likely than women to purchase supplements (OR = 2.047, *p* = .001). Factor 1 significantly predicted purchase (OR = 1.883, *p* < .001), whereas Factor 2 did not. A significant Sex × F1 interaction (OR = 0.475, *p* = .003) indicated that the positive association of F1 with purchase was stronger in women than in men (Table 3).

**Table 3:** Logistic regression (ORs with 95% CI).

Predictor	OR	95% CI	*p*
BMI	0.875	0.834–0.918	<0.001^*^
Training frequency	1.791	1.565–2.050	<0.001^*^
Diet	2.137	1.250–3.653	0.006^*^
Sex (male)	2.047	1.320–3.176	0.001^*^
F1	1.883	1.494–2.372	<0.001^*^
F2	0.980	0.787–1.220	0.855
Sex × F1	0.475	0.289–0.779	0.003^*^
Sex × F2	1.094	0.726–1.648	0.668

*Statistical significance at *p* < 0.05.

Receiver operating characteristic (ROC) curve for the final logistic regression model including BMI, training frequency, dieting status, sex, two motivation factors (F1 and F2), and their sex interactions (sex × F1, sex × F2). The model achieved excellent discrimination with an area under the curve (AUC) of 0.819. The dashed diagonal represents the line of no discrimination (AUC = 0.50).

### 3.3 Predictors of supplement spending

The linear regression model predicting log-transformed spending explained 8.6% of the variance. Higher scores on Factor 1 (*β* = –0.263, *p* < .001) and Factor 2 (*β* = –0.114, *p* = .004) were associated with lower spending per occasion. BMI (*β* = –0.113, *p* = .001) and training frequency (*β* = –0.073, *p* = .018) were also negative predictors, while diet and sex were not significant (Table 4).

**Table 4:** Standardized beta coefficients for spending.

Predictor	*β*	*p*
BMI	−0.113	0.001^*^
Training frequency	−0.073	0.018*
Diet	−0.003	0.933
Sex	0.025	0.446
F1	−0.263	<0.001*
F2	−0.114	0.004*

*Statistical significance at *p* < 0.05.

### 3.4 Mediation analysis

The indirect effect of BMI on spending through Factor 1 was small and not statistically significant (a × b = –0.0018, 95% CI: −0.00463 to 0.00091), indicating no evidence for mediation (Table 5).

**Table 5:** Mediation paths.

Path	Estimate	Bootstrap 95% CI
a (BMI → F1)	0.0091	–
b (F1 → log spending)	−0.1980	–
a × b (indirect)	−0.0018	−0.00463 to 0.00091

## 4 Discussion

In brief, higher training frequency, current dieting, male sex, and stronger health–appearance motivation were each associated with a greater likelihood of on-site purchase, whereas higher BMI was associated with lower purchase odds; the motivational association was stronger among women. Spending per occasion, by contrast, was modestly and negatively related to motivation, training frequency, and BMI, and no BMI → motivation → spending mediation was detected. We interpret these findings below, distinguishing what the data support from what remains speculative. Overall, the findings provided strong support for H1–H5, partial support for H6, and no support for H7.

### 4.1 Behavioral engagement and dieting as drivers of purchase

Consistent with H1, higher training frequency substantially increased the likelihood of supplement purchasing. In recreational fitness contexts, frequent training may normalize supplement use as part of a broader self-management routine (12). Dieting status independently predicted supplement purchasing, supporting H2. Because our dieting item captured only whether participants followed any kind of diet, without specifying its type or purpose, we interpret this association as a broad, undifferentiated signal rather than evidence about any particular dietary strategy. Findings from community- and family-based lifestyle interventions indicate that dietary behaviors are often embedded within broader health-management strategies (14, 15).

### 4.2 Motivational integration and BMI-related restraint

Support was observed for H3, as the health–appearance dimension (F1) emerged as one of the more robust predictors of purchase likelihood, consistent with Self-Determination Theory. We emphasize, however, that F1 is a data-derived dimension from five dichotomous items rather than a validated construct, so this interpretation should be regarded as provisional.

Higher BMI was associated with a lower likelihood of supplement purchasing, supporting H4. Because body image, appearance-related concerns, dietary restraint, and eating behavior were not measured in the present study, we cannot attribute this association to any of these mechanisms. One possible interpretation, which remains untested here and should be regarded as hypothesis-generating, is that individuals with higher BMI adopt more planned or restrained purchasing patterns. Equally plausible explanations include off-site or bulk provisioning, greater price sensitivity, or explicit dietary rules. BMI should not be read as a direct indicator of dietary intake, body composition, or muscle mass. Distinguishing among these accounts will require direct measurement of the relevant psychological and behavioral constructs in future work.

### 4.3 Sex moderation and identity-related processes

The significant Sex × F1 interaction indicates that the statistical association between the health–appearance dimension and purchase likelihood was stronger among women than among men. Our design does not identify the mechanism underlying this difference; because appearance motives, body image, and related constructs were not measured, mechanistic explanations specific to women would be speculative and are not drawn here. Prior work suggests that women more often endorse integrated appearance–well-being motives (10), and that among men motivational processes may be structured more around performance or athlete identity (16), but these accounts remain to be tested directly in relation to purchasing.

### 4.4 Purchase likelihood vs. spending magnitude

Results partially supported H6. While motivational and behavioral engagement factors predicted per-occasion spending, effect sizes were notably smaller than those observed for purchase likelihood. Higher motivation, higher training frequency, and higher BMI were all associated with lower spending per visit. This pattern suggests a “frequent but frugal” purchasing profile, in which motivated exercisers purchase supplements more often but select smaller, targeted products rather than engaging in higher discretionary spending.

This distinction between purchase likelihood and spending magnitude highlights the importance of conceptualizing these outcomes as related but distinct behavioral processes. Spending behavior appears to be more sensitive to situational constraints—such as pricing, timing, and contextual cues—than to stable motivational traits alone. Objective assessments of physical activity and functional engagement have similarly demonstrated that behavioral intensity does not necessarily translate into proportional increases in resource utilization (13).

### 4.5 Mediation analysis

The hypothesized mediation pathway from BMI to spending via health–appearance motivation (H7) was not supported. Although BMI exhibited a modest association with motivation and motivation predicted spending, the indirect effect was not statistically significant. This finding suggests that BMI-related differences in spending are likely driven by mechanisms not fully captured by the present motivational framework, such as dietary restraint, financial planning, or explicit food rules. Incorporating these constructs in future research may help clarify the pathways linking anthropometric status and consumption behavior.

### 4.6 Integrative interpretation

Taken together, the findings suggest that supplement purchasing in recreational fitness centers reflects a complex interplay of behavioral commitment, motivational integration, anthropometric status, and sex-specific psychological processes. Individuals who are highly engaged and internally motivated are more likely to purchase supplements, yet they tend to spend less per occasion, indicating structured, goal-congruent consumption rather than impulsive behavior. Higher BMI appears to be associated with restraint rather than increased consumption, and motivational influences operate differently across sexes.

### 4.7 Practical implications

These findings carry several implications for fitness center operators and health promotion initiatives.

First, motivationally aligned messaging may enhance engagement. Framing on-site products as recovery-supportive or goal-consistent may resonate particularly with individuals high in integrated health–appearance motivation.

Second, the inverse association between BMI and spending suggests that restrictive or weight-management-oriented individuals may benefit from structured, transparent, and lower-calorie options that reduce perceived conflict between goals and convenience.

Third, sex-sensitive approaches may improve targeting strategies. Messaging emphasizing wellbeing and functional recovery may resonate more strongly with women, whereas muscularity-specific cues may be more effective for certain male subgroups.

Importantly, any such strategies should prioritize evidence-based supplementation and avoid reinforcing harmful body image norms.

### 4.8 Strengths and limitations

Strengths include the objective, on-site measurement of height and weight—an improvement over the self-reported anthropometry common in supplement-use research—together with the combination of behavioral, anthropometric, and exploratory motivational variables within a single multivariable framework. We report the achieved sample size neutrally (*N* = 1,087); no *a priori* power analysis was conducted, and we therefore avoid describing the sample simply as “large.” We do not claim BMI itself as a strength, given its acknowledged limitations as an anthropometric construct.

Several limitations should be acknowledged. (i) BMI is an indirect indicator of weight status that does not differentiate fat mass from lean mass; interpretations linking BMI to psychological or dietary mechanisms are necessarily speculative because these constructs were not assessed. (ii) Dieting status was a single yes/no item that did not capture diet type, purpose, professional supervision, nutritional knowledge, or exposure to nutrition-related marketing and social media. (iii) Although data were drawn from two facilities rather than one, both belong to a single commercial chain and a single region of Hungary; the sample reflects the cultural, geographical, and socioeconomic characteristics of urban and small-city recreational exercisers in South Transdanubia and may not generalize to other operators, regions, or countries. (iv) The motivational dimensions were derived from only five dichotomous items rather than a validated instrument, so their construct validity is uncertain and interpretations built on F1 should be regarded as provisional. (v) Model discrimination was assessed on the fitting sample only; without cross-validation, bootstrap correction, or an independent cohort, the AUC may overstate out-of-sample performance. The cross-sectional design also precludes causal inference, and spending was derived from categorized self-reports. Future studies should employ longitudinal designs, validated multi-item motivational instruments, objective transaction-level data, and internal/external validation of prediction models.

## 5 Conclusion

Dietary supplement purchasing behavior in recreational fitness environments emerges from the interaction of anthropometric status, behavioral investment, and motivational structure rather than from performance considerations alone. Higher training frequency, ongoing dieting, male sex, and integrated health–appearance motivation increased the likelihood of purchase, whereas higher BMI was consistently associated with lower purchasing probability and spending. The divergence between purchase frequency and spending magnitude suggests that motivated exercisers may adopt structured, goal-consistent micro-consumption patterns. Given the exploratory nature of the motivational dimensions and the single-chain sampling frame, these conclusions should be regarded as a basis for confirmatory, multi-site research rather than as definitive causal claims.

## Data Availability

The raw data supporting the conclusions of this article will be made available by the authors, without undue reservation.
